# Development and treatment of spinal deformity in patients with cerebral palsy

**DOI:** 10.4103/0019-5413.62052

**Published:** 2010

**Authors:** Athanasios I Tsirikos

**Affiliations:** Clinical Lead-Scottish National Spine Deformity Center, Royal Hospital for Sick Children, Sciennes Road, Edinburgh, EH9 1LF, UK

**Keywords:** Scoliosis, cerebral palsy, surgery, spinal fusion, outcome

## Abstract

Scoliosis is a common deformity in children and adolescents with cerebral palsy. This is usually associated with pelvic obliquity due to extension of the curve to the sacrum. Sagittal plane deformity is less common and often develops along with scoliosis. Spinal deformity in patients with severe neurological handicaps can affect their ability to sit and cause significant back pain or pain due to rib impingement against the elevated side of the pelvis on the concavity of the curvature. Surgical correction followed by spinal arthrodesis is indicated in patients with progressive deformities which interfere with their level of function and quality of life. Spinal deformity correction is a major task in children with multiple medical co-morbidities and can be associated with a high risk of complications including death. A well-coordinated multidisciplinary approach is required in the assessment and treatment of this group of patients with the aim to minimize the complication rate and secure a satisfactory surgical outcome. Good knowledge of the surgical and instrumentation techniques, as well as the principles of management is needed to achieve optimum correction of the deformity and balancing of the spine and pelvis. Spinal fusion has a well-documented positive impact even in children with quadriplegia or total body involvement and is the only surgical procedure which has such a high satisfaction rate among parents and caregivers.

## INTRODUCTION

Cerebral palsy (CP) is a static encephalopathy, which affects the immature brain and results in permanent motor disability and frequently the development of a spinal deformity. CP is described by the pattern of motor disorder as spastic, flaccid, athetoid and mixed type with spastic and mixed accounting for 85% of all patients.^1^ In terms of anatomical pattern of body involvement, it can be divided into monoplegia, diplegia, hemiplegia, triplegia, and quadriplegia. Total-body involvement refers to a patient with severe quadriplegia combined with marked mental disability.

Spinal deformity in growing patients with CP develops due to the combination of spasticity, muscle weakness, as well as incompetent muscle control; this can result in poor trunk balance and significantly limits the patient's ability to function. The overall incidence of spinal deformity in CP has been reported at 20-25% ranging from 5% in spastic diplegia to 74% in spastic quadriplegia and this is directly proportionate to the degree of neurological deficit, as well as the ambulatory capacity.^2^–^11^

The development of trunk imbalance along with the associated pelvic obliquity can affect both standing balance and walking ability in ambulatory patients. It may also cause sitting intolerance in wheelchair-bound patients due to collapse of the spine and painful costopelvic impingement on the concavity of the scoliosis and these patients are gradually converted into hand-dependent sitters.^8^,^9^ Patients with a severe scoliosis can develop cardiopulmonary complications due to chest distortion which follows the spinal deformity, as well as pressure sores in the buttock area due to asymmetrical loading in the presence of skin insensitivity and pelvic decompensation.^11^ Associated medical co-morbidities such as gastro esophageal reflux, swallowing disorders, repeated aspiration may also get aggravated with progression of the spinal deformity.

The purpose of this review is to present the characteristics and specific considerations of spinal deformity in children and adolescents with CP, as well as provide guidelines for treatment of these complex deformities in the presence of severe associated medical co-morbidities, which can significantly compromise the surgical outcome. The treatment algorithm applying a standardized perioperative protocol is presented and operative techniques using segmental spinal instrumentation with sub laminar wire or pedicle-screw constructs are discussed.

## TYPES OF DEFORMITY

Scoliosis often associated with increased thoracic or thoracolumbar kyphosis is the most commonly encountered deformity in patients with CP.^12^ Lumbar lordoscoliosis, one of the most difficult deformities to correct surgically, occurs less frequently. In young children, a flexible postural curvature develops and this becomes structural with further growth and as the patient approaches puberty. Puberty may begin earlier or often later in a patient with CP compared to unaffected individuals.^4^,^6^,^13^,^14^ The rate of progression of the scoliotic curve during the adolescent growth spurt increases to 2 - 4° / month and a rigid deformity develops rapidly, especially in patients with quadriplegia or total body involvement CP.^15^ ^16^ Scoliosis continues to progress beyond skeletal maturity at a rate of 1.4 − 4° / year if the curvature is greater than 50° at the end of growth compared to 0.8° / year for curves of less than 50°.^13^ ^14^ ^17^

Two scoliotic patterns have been described in patients with CP.^5^ Group 1 curves develop in patients with good ambulatory function and less severe neurological deficit such as patients with monoplegia or hemiplegia. They resemble idiopathic scoliosis and involve single thoracic or thoracic and lumbar curves with level pelvis.^5^ Their treatment follows similar criteria to those applied in the management of idiopathic scoliosis and requires a spinal arthrodesis short of the sacrum. Group 2 curves are typically seen in more severely affected patients who are wheelchair dependent and include long thoracolumbar or lumbar C-shaped collapsing curves associated with pelvic obliquity. Group 2 curves can be subdivided into 2A if the sacrum is part of the scoliosis and 2B when the sacrum does not form part of the curve.

Pelvic obliquity may develop due to extension of scoliosis distally to include the sacrum and the pelvis or due to ‘wind- swept’ deformity of the hips resulting from asymmetrical contractures of the hip abductors and subsequent hip imbalance.^18^ A combination of both factors is often present. Previous reports have suggested that adductors’ contracture and hip subluxation may lead to pelvic obliquity, which is followed by the development of scoliosis.^9^ In the presence of a collapsing scoliosis with significant pelvic obliquity, as well as associated hip subluxation or dislocation, it is usually best to treat the spinal deformity first with the aim to stabilize the spine, level the pelvis and facilitate relocation of the hips once the pelvic deformity is been corrected.

Isolated sagittal spinal deformity in patients with CP is rare but it can coexist with scoliosis with a reported prevalence of 7%.^19^,^20^ In the author's experience, a long thoracolumbar kyphosis producing a collapsing spine with a positive sagittal balance is very commonly present in association with a scoliosis in patients with quadriplegia. Patients with generalized trunk hypotonia, hamstring spasticity or fixed hamstring contractures causing posterior pelvic inclination and decreased lumbar lordosis also develop a compensatory thoracic hyperkyphosis. Children who have undergone dorsal rhizotomy through extensive laminectomies tend to develop a thoracolumbar kyphosis. The sitting ability and head control in a functional position become affected as a result of the kyphotic deformity and global sagittal spinopelvic imbalance.

Increased lumbar lordosis is less common but it may develop in ambulatory patients with diplegia who have flexion contractures of the hips and an anterior pelvic tilt. An isolated lumbar lordosis may also develop following multi-level laminectomies for dorsal rhizotomy or rarely as the result of tethering of the spinal cord. Children with lumbar hyperlordosis have an increased risk of developing isthmic spondylolysis and spondylolisthesis and often complain of low back pain.

## TREATMENT OF SPINAL DEFORMITY

The objective of management of spinal deformity in CP is to maintain or improve the patients' functional abilities, as well as quality of life. The decision-making for treatment must be tailored to the individual patient and should be based on a meticulous risk-benefit assessment depending on the severity of co-existing medical morbidities.

### a. Conservative treatment

This includes seating adaptations as well as the use of spinal braces although none of these measures can prevent curve progression or alter the natural history of the deformity. Seating supports are useful to improve trunk balance, maintain an upright posture and facilitate patient care.^21^,^22^ These include an adjustable head support, offset chest lateral rests, shoulder harnesses and straps, and a waist strap all fitted on the wheelchair, as well as the ability of the wheelchair to tilt in space and accommodate for the patient's deteriorating posture in the course of the day. Molding of the back of the seat may be needed for patients with terminally fixed and rigid deformities but this is contraindicated in growing children with deteriorating deformities.

Brace treatment using a molded thoracolumbosacral orthosis (TLSO) can be applied in young patients with small and flexible curves in an attempt to slow down deformity progression, support the spine, preserve vertebral growth, and delay surgical correction for as late as possible.^23^–^25^ No impact of the spinal brace on the rate of scoliosis deterioration, or the natural history of the deformity has been previously documented,^15^ even though a recent study has supported a possible benefit of bracing in certain patients.^26^ The tight fitting of a conventional spinal brace may cause skin irritation, respiratory compromise, exacerbation of gastro esophageal reflux and feeding / swallowing disorders, as well as lead to poor patient compliance. A soft bi-valved whole-body brace is usually better tolerated by patients with impaired neurological control; a combination of such brace when wheelchair is not used along with seating adaptations on the wheelchair is probably the most appropriate temporizing measure for young patients with flexible curves.

### b. Surgical correction and spinal arthrodesis

Surgical correction is the only effective management of severe deformities in patients with CP and this has a well- documented positive impact in the patients’ quality of life. The aims of the surgery are to balance the spine and pelvis, restore trunk alignment, prevent progression of the deformity, improve sitting/standing posture and walking ability, prevent respiratory, feeding and skin complications, as well as maximize overall level of function. 2,22 A successful surgical outcome would produce a balanced spine in the coronal and sagittal planes with level shoulders and pelvis, as well as the chest centered on top of the pelvis.^1^

The indications for surgical correction include: a) Scoliosis that exceeds 45-50° in children 10 years of age or older, b) Curve progression of greater than 10°, or c) Significant deterioration in the child's ability to function.^3^ ^5^ ^22^ Patients with very severe and rigid curvatures are at a greater risk of developing serious postoperative complications that can be life-threatening in view of their associated medical co-morbidities, as well as the extent of surgery. Scoliosis correction in the presence of such extreme deformities may be very limited even with a combined anterior-posterior spinal approach; the risks of surgery in this group of patients, therefore, outweigh any possible benefits and they should be considered poor candidates for deformity correction. In contrast, spinal surgery would be recommended in patients with a scoliosis of approximately 40° at skeletal maturity due to the risk of further deterioration into adult life.

## PREOPERATIVE EVALUATION/MEDICAL CO-MORBIDITIES

A multidisciplinary approach is essential during preoperative assessment with the aim to select candidates who are more likely to benefit from surgical correction of their deformity. Due to a host of medical co-morbidities that need to be investigated, the multidisciplinary team needs to include anesthetists, neurologists, respiratory physicians, cardiologists, gastroenterologists, dieticians, physiotherapists, occupational therapists, and intensive nursing care.

### a. Respiratory

Postoperative chest infection, as well as respiratory failure may occur much more frequently in patients with CP undergoing spinal surgery compared to those with an idiopathic scoliosis.^27^ These children may have abnormal hypo pharyngeal tone or anatomical abnormalities affecting their upper airway, as well as a poor coughing mechanism due to weak respiratory muscles.^28^ The risk of postoperative pneumonia can be significantly increased following an anterior approach to the thoracic or thoracolumbar spine. Aggressive chest physiotherapy, as well as potentially non- invasive ventilation and a cough-assist machine should be routinely instituted following surgery and until the patient is medically stable.

Gastro esophageal reflux and a poorly coordinated swallowing mechanism can be suspected from the history of coughing episodes during feeding, as well as the development of recurrent aspiration pneumonias, which may lead to progressive lung damage and fibrosis.^28^ Preoperative chest radiographs may show evidence of pulmonary fibrosis and lung function tests would detect respiratory compromise; however, these are often impossible to perform due to poor patients' cooperation. Episodes of prolonged and recurrent oxygen desaturations during sleep indicate poor respiratory reserve. The reduction in the anteroposterior diameter of the chest secondary to thoracic lordosis can cause bronchial obstruction, which in conjunction with the decrease in lung volume due to the scoliosis will further impair gas exchange. Prolonged postoperative care in the intensive care unit (ICU) may be anticipated in selective patients even though prolonged ventilation post-surgery is rarely required. Placement of a tracheostomy postoperatively may be necessary in rare occasions to assist weaning the patient off the ventilator and aggressive pulmonary toilet can prevent the risk for chest infection. Preoperative tracheostomy is only indicated in children with complex upper airway abnormalities.

### b. Nutritional/immunological

Global developmental delay is common in patients with CP and affected children are usually below the 25^th^ or even the 5^th^ percentile for age on the growth charts. Oral feeding and swallowing may be a problem due to uncoordinated muscle contraction or abnormal pharyngeal muscle tone. The nutritional status of the patients must be evaluated with a thorough history and clinical examination, as well as hematological and biochemical tests. Poor nutritional status predisposes the patients to delayed wound healing and a poor immunological response to infection. A significantly lower infection rate, a shorter period of endotracheal intubation and less hospitalization time after spinal arthrodesis has been reported in CP patients with a preoperative serum albumin of 35 g / L or above and a total blood lymphocyte count of 1.5 cells × 10^9^/L or above.^29^

Surgical intervention should be postponed until the patient is nutritionally adequate with normal serum albumin levels. Feedings through a nasogastric or gastrostomy tube can optimize nutritional state before surgery is scheduled. Gastritis, peptic ulcers, as well as gastrointestinal dysmotility predispose to severe gastro esophageal reflux and constipation in these patients. Nissen fundoplication may improve the symptoms of reflux. However, often deformity correction also has a positive impact on preexisting reflux and, therefore if the reflux is not severe it might be best to delay surgical repair until after spinal fusion. Bowel preparation allows early reestablishment of feedings and prevent overflow diarrhea. A long general anesthetic using opioids, as well as prolonged postoperative pain management would increase the risk of developing an ileus.^28^ Parenteral nutrition may be needed if there is persistent paralytic ileus and nutritional malabsorption.

Patients with CP may develop asymptomatic chemical pancreatitis diagnosed by an increase of the serum amylase after spinal arthrodesis. Superior mesenteric artery syndrome may occur when significant correction of severe scoliotic curvatures is achieved, particularly in the presence of associated lumbar hyperlordosis.^1^

### c. Intraoperative blood loss

Blood loss can be high during spinal arthrodesis in children with CP. These patients have inherent or acquired disorders of coagulation and are on anti-seizure medications, such as sodium valproate or phenobarbital, which increase the risk of severe perioperative bleeding.^30^ It is possible that these medications may be discontinued and an appropriate alternative commenced prior to surgical intervention. Tsirikos *et al*.^31^,^32^ report a mean intraoperative blood loss of 2.9 L, corresponding to 1.2 blood volumes in a review of 288 consecutive patients undergoing scoliosis correction. A minimum one-blood volume of blood must be typed and cross-matched for spinal surgery and fresh frozen plasma with clotting factors should be readily available. The intraoperative use of cell saver, as well as controlled hypotensive anesthesia along with muscle relaxation and meticulous hemostasis during tissue dissection can reduce blood loss and the need for transfusion. The problems associated with large-volume blood transfusions need to be treated early in order to prevent anaphylaxis, electrolyte imbalance and disseminated intravascular coagulation.

### d. Behavioral/neurological

Mental retardation, learning difficulties and behavioral disorders may complicate the postoperative care of these patients, as good compliance is necessary for chest physiotherapy, mobilization, toileting, as well as feeding. Patients with ventriculoperitoneal shunts should be assessed before and after spinal surgery to confirm adequate function of the shunt. Intrathecal baclofen therapy has been introduced to control severe spasticity refractory to oral anti-seizure agents. Shah *et al*.^33^ have shown that the administration of botulinum toxin through an intrathecal pump in patients with CP does not cause progression of their scoliosis, which simply follows the natural history of the deformity. During scoliosis surgery, the intrathecal catheter of the Baclofen pump has to be preserved in situ during the posterior exposure to the spine or it can be repositioned at the end of the procedure.

### e. Metabolic

Poor bone quality due to disuse osteopenia in wheelchair- bound patients, as well as antiepileptic medication induced osteomalacia increases intraoperative blood loss, as well as the risk of instrumentation failure. Preoperative administration of intravenous bisphosphonates has been considered, particularly in quadriplegic patients with no ambulatory function and marked osteopenia in order to optimize their bone quality before spinal deformity surgery.^1^

### f. Risk of infection

Patients with severe CP have a significant risk of infection due to inherent immunodeficiency aggravated by poor nutrition. Apart from pulmonary infection they can also develop urine or deep wound infection in the postoperative period and this can threaten the surgical outcome. The presence of an active urine infection can increase the risk of a deep wound infection. Patients who are double incontinent have a significant risk for developing deep wound infections due to direct contamination.

## SURGICAL CORRECTION TECHNIQUES

The advent of Harrington instrumentation significantly advanced the surgical treatment of neuromuscular scoliosis; however, the distraction techniques applied were difficult and often impractical in patients with quadriplegia and osteopenic vertebrae.^34^ In addition, the recorded rate of non-union following spinal arthrodesis, as well as the subsequent deformity progression remained high.^35^–^37^

Luque (1977)^35^ introduced the concept of segmental spinal fixation with the use of sub laminar wires which distributes the corrective forces widely over each instrumented vertebra, allows for a greater degree of deformity correction and provides increased initial stability, thus, reducing the risk of instrumentation failure and pseudarthrosis.^2^ ^38^ ^39^ The laminae provide the strongest point of fixation compared to the pedicle or the vertebral body in patients with CP who have poor bone quality and can withstand segmental translational forces applied through the sub laminar wires in order to achieve correction of the deformity and balancing of the spine in both coronal and sagittal planes. Allen and Ferguson^40^ developed the Galveston technique of intramedullary placement of the Luque rods within the iliac beds with the aim to achieve secure pelvic fixation and allow for a lumbosacral fusion in patients with severe associated pelvic deformity. However, initial enthusiasm vanished due to reports of a rate of non-union using the Luque system up to 10%, instrumentation-related complications up to 21%, as well as curve progression after initial arthrodesis in 30% of patients.^9^,^41^,^42^ The high incidence of pseudarthrosis was due to the use of two unconnected soft rods which were moving independently and failed to provide stable spinal fixation even when postoperative immobilization was used.^42^

**Figure 1:**
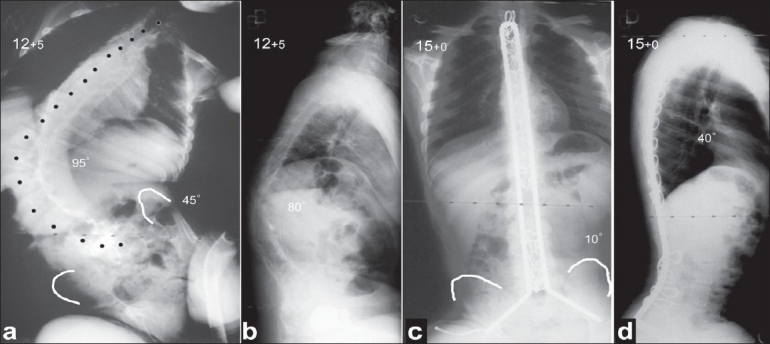
Preoperative posteroanterior (a) and lateral (b) radiographs of the spine show a severe collapsing kyphoscoliosis with associated pelvic obliquity and rib impingement. Radiographs (c, d) taken 2.5 years following a posterior spinal fusion using the Unit rod technique show an excellent balance of the spine in the coronal and sagittal planes and a nearly level pelvis. Note the presence of dextrocardia

The Unit rod instrumentation followed the principles of the Luque-Galveston technique but incorporated a single U-shaped rigid rod which allowed for stable segmental fixation of the spine and pelvis resulting in an improved correction of the spinal and pelvic deformity, as well as restoration of coronal and sagittal trunk balance [Figure 1].^43^,^44^ In the largest reported series of 287 children and adolescents with severe CP who were treated for their spinal deformity using the Unit rod technique, an excellent correction of the scoliosis to 68% and of the pelvic obliquity to 71% was achieved with good lateral balance of the spine and a relatively low rate of complications at a mean postoperative follow-up of 3.2 years.^32^ In the author's opinion, the Unit rod remains the primary system for the treatment of patients with CP because it is simple to use, is considerably cheaper compared to all pedicle screw or pedicle screw/hook instrumentation and can achieve excellent deformity correction with a low loss of correction and re-operation rate at follow-up, as well as a low prevalence of associated complications.

**Figure 2:**
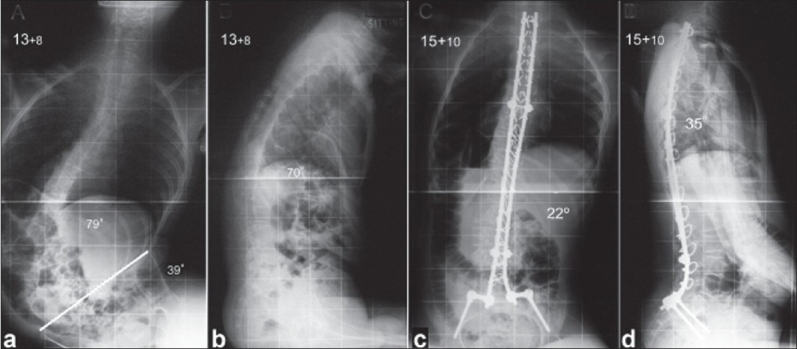
Preoperative posteroanterior (a) and lateral (b) radiographs of the spine show a severe kyphoscoliosis with collapsing of the spine against the elevated right side of the pelvis. Follow-up radiographs (c, d) 2.1 years after a posterior spinal fusion with the use of two rigid connected rods as well as pelvic fixation through iliac bolts show excellent correction of the deformity and a balanced spine and pelvis

An alternative to Unit rod instrumentation is the use of two rigid rods with proximal and distal cross-connectors secured to the spine by segmental sub laminar wires, at every level from the proximal thoracic spine to L5. Iliac bolts can be used bilaterally and these should be connected to the distal end of the rods in order to prevent mobility across the lumbosacral junction and allow for a solid fusion [Figure 2]. The convex rod is positioned first and is used for deformity correction while the concave rod is supportive in order to augment stability of the construct. In the author's experience, the use of these connected rigid rods with secure distal screw fixation into the pelvis can achieve comparable deformity correction to the Unit rod technique.

**Figure 3:**
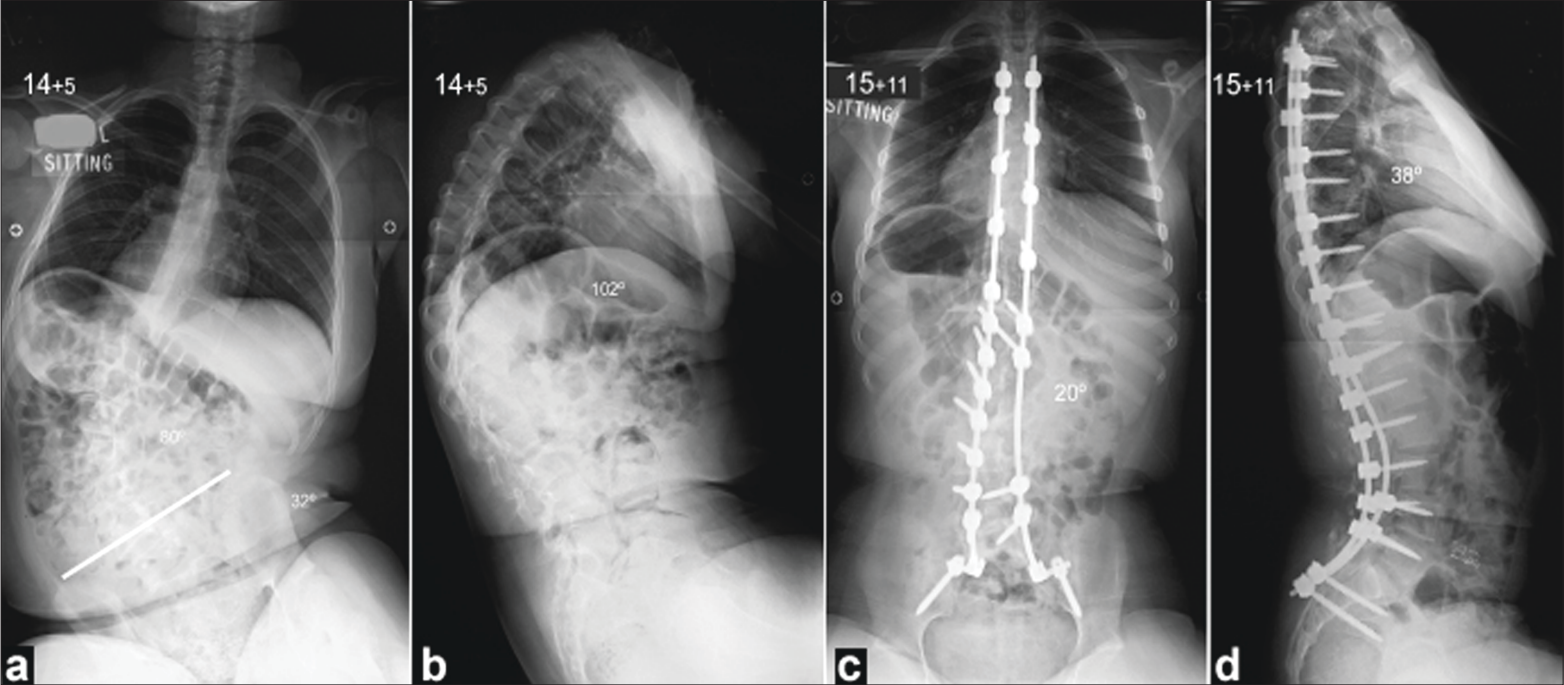
Preoperative posteroanterior (a) and lateral (b) radiographs of the spine show a severe kyphoscoliosis with associated pelvic obliquity on the right side, as well as rib impingement. The patient underwent a posterior spinal fusion with the use of pedicle screw instrumentation (c, d), which resulted at an excellent correction of the deformity as well as a balanced spine with level pelvis

Third generation instrumentation uses all pedicle screw or hybrid constructs (combination of hooks and screws) and has been popularized in the treatment of idiopathic scoliosis; it was recently introduced in the surgical correction of neuromuscular scoliosis.^45^,^46^ This follows the Luque principle of segmental spinal fixation allowing for gradual correction of the deformity along multiple levels and can be combined with iliac bolts for lumbo-pelvic or sacro-iliac plates for sacro-pelvic fixation [Figure 3]. In patients with marked osteopenia, a pedicle screw or hybrid system may fail to provide as stable vertebral column fixation as a sub laminar wire instrumentation and this would limit the ability to perform corrective maneuvers and increase the risk of implant failure and pseudarthrosis. Third generation instrumentation is also significantly more expensive compared to the Luque system or the Unit rod without any so far documented advantage in terms of deformity correction or associated complications compared to the Unit rod, which still remains the gold standard technique in patients with CP. In contrast, if repeat surgery is required (for example, in case of a non-union), it is technically easier and neurologically safer to revise an instrumentation system which consists of pedicle screws or hooks compared to sub laminar wires. Two series presented by Teli *et al*.^45^,^46^ from two different spinal centers included 60 and 36 patients with CP treated with posterior or anteroposterior spinal arthrodesis using a hybrid instrumentation construct; the authors reported scoliosis correction of 53-65%, as well as pelvic obliquity correction of 40-50% with a prevalence of major complications of 13.5% and 10.7% respectively. These results indicate a lesser degree of spinopelvic deformity correction but a similar rate and type of complications compared to the Unit rod system.^32^,^45^,^46^

In the author's practice, the use of an all pedicle screw construct compared to the Unit rod technique requires less surgical time as the placement of pedicle screws in experienced hands is significantly faster than the placement of sub laminar wires. The reduction in surgical time results in less intraoperative blood loss, which is further reduced due to the fact that the spinal canal is not exposed and the epidural vessels are not injured as often when sub laminar wires are placed segmentally. The pedicle screw technique can also be performed with a lesser need for experienced assistance whereas any technique involving placement of sub laminar wires requires two experienced and adequately trained surgeons.

## SURGICAL CONSIDERATIONS

### a. Levels of fusion

In Group 1 curves, it is indicated to fuse the curvature short in order to preserve distal mobile spinal segments following the principles that apply in the treatment of idiopathic scoliosis.^3^ In contrast, typical long C-shaped thoracolumbar or lumbar curves with pelvic obliquity require extension of the spinal arthrodesis from the upper thoracic spine to the sacrum with pelvic fixation in order to restore balance of the trunk.^2^,^22^ Extending the arthrodesis usually to T2 is more likely to prevent the development of junctional kyphosis above the most cephalad instrumented level which is a common problem in patients with poor muscle control.^22^,^47^ Short spinal arthrodesis ending in the lumbar region and sparing the lumbosacral junction has been associated with a high risk of distal add-on deformity leading to reoperation.^3^,^22^,^43^

Earlier attempts to correct scoliosis in patients with CP using the Harrington instrumentation often created an iatrogenic flat back deformity or required long periods of bed rest and body casting to achieve a fusion; these were both factors adversely affecting long-term the patient's walking function. Therefore, traditionally fusion to the pelvis has been contraindicated in patients with good ambulatory capacity.^2^,^5^,^22^,^47^,^48^ Tsirikos *et al*.^49^ have shown that fusion to the sacrum with bilateral pelvic fixation using the Unit rod system can restore coronal and sagittal balance of the spine and allow for early patient mobilization without compromising the ability to ambulate. In addition, in the author's experience, quadriplegic patients who lost their walking ability due to the development of severe collapsing scoliosis often regain satisfactory ambulatory function following a successful long fusion to the sacrum and pelvis.

### b. Surgical approach

The majority of pediatric patients with CP and spinal deformities can be treated through a posterior spinal arthrodesis. An additional anterior spinal release is indicated in the presence of rigid curves in order to maximize scoliosis flexibility and improve the ability to correct the deformity using instrumentation. Longitudinal traction radiographs with the patient in the supine position or suspension posteroanterior views of the spine are useful to assess curve stiffness and determine the need for anterior surgery. A combined anterior release-posterior instrumented fusion is indicated when the scoliosis cannot be corrected to 40-50°, when there is fixed pelvic obliquity of more than 10° or when the thorax cannot be balanced over the pelvis.^2^ Side-bending radiographs are not reliable due to poor patient compliance. Radiographs with the patient in trunk hyperextension against the bolster can be obtained to evaluate flexibility of kyphotic deformities. Clinical evaluation of the rigidity of the curve can also be achieved using the physical examination side-bending test introduced by Freeman Miller^50^ with the patient bent against the examiner's knee.

The anterior procedure should include release of the anterior longitudinal ligament, complete annulectomy and discectomy back to the posterior longitudinal ligament, as well as excision of the vertebral end plates in order to provide angular and rotational mobility of the spinal segments at the apex of the deformity. This can produce better curve correction, as well as a circumferential spinal fusion, which will minimize the risk of pseudarthrosis and recurrence of the deformity.^5^,^9^,^19^,^22^,^36^,^39^,^51^ It can also prevent the risk of crankshaft effect due to continuous anterior vertebral growth in the presence of a solid posterior fusion in young patients, which could not be avoided with the use of the Luque rods and would lead to progressive deformity.^9^ ^42^ Smucker and Miller^52^ followed 29 children with open triradiate cartilage who underwent posterior spinal arthrodesis with the Unit rod until skeletal maturity and reported no progression of the curvature during the remaining stages of growth. In the author's experience, the use of a pedicle screw construct, which allows for segmental fixation of all three spinal columns can also arrest anterior vertebral growth and prevent the development of crankshaft without the need for an additional anterior release.

When an anterior release is needed, the use of anterior instrumentation can increase spinal stability and enhance interbody fusion. However, in the author's opinion, this is mostly recommended if the anterior instrumented arthrodesis can produce a nearly straight segment of the spine; this will still need to be supplemented by a longer posterior instrumented arthrodesis in order to correct the whole length of the spinal deformity and level the pelvis.

There is considerable debate on whether the anterior and posterior spinal procedures in patients with neuromuscular scoliosis should be staged. Most of the reports in the literature favor the single-stage procedures, however, they refer to mixed populations of patients with different underlying neurological diagnosis.^53^–^58^ Tsirikos *et al*.^49^ have shown in an isolated group of patients with CP that one and two-stage anteroposterior spinal arthrodesis can produce comparable results in terms of deformity correction; however, in their series, the same-stage procedures were associated with a considerable mortality rate including three immediate postoperative deaths, increased surgical morbidity, as well as a higher rate of technical complications. In the author's practice, staging the spinal procedures under separate anesthetics, five to seven days apart, is safer in patients with severe deformities and concomitant medical illness.

### c. Sagittal deformity

Sagittal deformity occurs commonly in association with scoliosis in children with CP. Thoracic hyperkyphosis is often associated with hamstring tightness and this can be improved following hamstring lengthening at an early age. In contrast, an established kyphotic deformity can be addressed through a posterior instrumented spinal arthrodesis, especially in the presence of patient's symptoms or impairment of function. This should extend from the cervicothoracic junction to the pelvis in order to prevent proximal junctional kyphosis and control posterior pelvic tilt. An additional anterior release may be required in the presence of a rigid kyphotic deformity. Excessive kyphosis may affect venous return to the heart and attention must be paid during surgery to maintain hemodynamic stability while correcting the deformity. Tsirikos *et al*.^31^ defined preoperative thoracic hyperkyphosis as the only deformity factor adversely affecting survival rates and statistically correlating with a poor life expectancy after spinal fusion in children with CP.

Lumbar hyperlordosis secondary to anterior pelvic tilt due to hip flexion contractures can be initially addressed with muscle lengthening. Fixed lordotic curves require anterior closing wedge osteotomies to increase flexibility and allow for correction through the posterior instrumented fusion. The coexistence of scoliosis and lumbar hyperlordosis makes the use of posterior instrumentation technically difficult and increases the incidence of implant fixation problems, as well as intraoperative morbidity.^32^ The development of significant anterior pelvic tilt can increase the risk of inner iliac table penetration and injury to the abdominal organs during placement of the pelvic legs of the Unit rod or whilst bending the rod in situ to correct the deformity in children who already have poor bone quality. The rate of reported complications involving pelvic fixation of the Unit rod is 15.1% with a preoperative lumbar lordosis of more than 60° as compared to 3.6% for a lordosis of less than 60° with associated increased intraoperative blood loss and prolonged surgical time.^32^ Iliac bolts are easier and safer to insert compared to the Unit rod in this group of patients and can be connected through the rods to pedicle screws in the lumbar spine which provide bony fixation and better correction of the lordosis, especially in patients who have undergone extensive laminectomies for dorsal rhizotomies.

Lipton *et al*.^59^ suggested that children with isolated sagittal curves of 70° or above are more likely to develop severe symptoms due to the deformity including two of their patients with hyperlordosis who developed superior mesenteric artery syndrome and one patient presenting with bowel and bladder dysfunction. In their series of 24 patients, deteriorating sitting balance, as well as back pain was the primary indication for surgical correction.

## TREATMENT ALGORITHM

In the author's practice, preoperative evaluation includes routinely a respiratory, cardiac and anesthetic assessment with the aim to determine the patient's fitness to undergo spinal deformity surgery. Sleep studies, an ECG and cardiac ultrasound are performed and the patients are reviewed in a joined pre-assessment clinic. The results of the tests are subsequently discussed at a multidisciplinary meeting with all specialties involved and the final decision for treatment is made. Nutritional disorders are addressed by the gastroenterology and dietetic teams; epilepsy or other neural-related issues (intrathecal baclofen pumps, ketogenic diet to treat refractory seizures, vagal nerve stimulators, and severe dystonia) have to be discussed with the pediatric neurologists. A bowel preparation is also indicated before surgery in patients who suffer from chronic constipation and who are at high risk of overflow diarrhea which can contaminate the spinal wound in the postoperative period.

Intraoperatively, all patients receive prophylactic antibiotics with the administration of a first-generation cephalosporin immediately before and for 24 hours after spinal surgery. Before anesthesia induction, arterial and central venous lines are placed, and the central venous line is maintained until the second stage in patients who will have a two-stage procedure. A nasogastric tube is used to decompress the stomach and a Foley catheter to monitor urine output. Cell- saver is used during the procedure to reduce the need for allogenic blood transfusion.

Spinal cord monitoring with the use of somatosensory or motor-evoked potentials is used during surgery only in ambulatory patients. Extensive facetectomies are always necessary in order to mobilize the curvature; a meticulous arthrodesis technique is performed across the facet joints followed by decortication of the posterior bony elements where abundant locally harvested autologous and allograft bone is placed to achieve a solid fusion. A wound drain is placed above the lumbosacral fascia and this is maintained for three to four days following spinal arthrodesis.

Postoperatively, the patients are transferred to the ICU, electively ventilated for a period of 24-48 hours until they are medically stable. This period gives an opportunity to restore hemodynamic, electrolyte and fluid balance, as well as adequately address postoperative pain. Non- invasive ventilation may be required for patients with poor respiratory reserve in order to optimize pulmonary recovery. Feedings are started soon in the postoperative period and these are usually delivered through a nasogastric, nasojejunal or gastrostomy tube in order to reduce the risk of gastrointestinal complications. If there is contraindication to enteral feedings, total parenteral nutrition can be used to reduce the risk of poor wound healing and infection.

Intensive physiotherapy is offered to clear chest secretions, prevent or treat respiratory infections and rapidly mobilize the patients out of bed. The patients' wheelchair needs to be assessed and modified in order to accommodate for their corrected spinal posture and seating balance after surgery. A reclining wheelchair can be used initially to provide better sitting comfort during the immediate postoperative period and while a patient who has been fused distally to the sacrum and the pelvis has difficulties to sit to 90°. No postoperative immobilization or external support is used.

Combined anterior and posterior spinal arthrodesis is electively performed under separate anesthetic sessions five to seven days apart. The patients are kept in the ICU between the two stages of the procedure under close medical care. The chest drain placed during the thoracotomy or thoracoabdominal approach to the spine is removed when the drainage is less than 100 ml/day, usually on the third or fourth day after surgery.

The patient should be ready for discharge when the surgical wound is completely healed, there is no sign of infection, sitting in his wheelchair is comfortable, and adequate feedings have been established. This occurs usually 10-15 days after spinal deformity surgery. Full recovery should be anticipated by six months following spinal arthrodesis.

## PREDICTORS OF POOR SURGICAL OUTCOME

The best predictor for the development of postoperative complications following spinal arthrodesis in children with CP is the degree of neurological disability.^11^ In the same study, patients with poor preoperative nutrition, gastrostomy feedings, anti-seizure medication, tracheal abnormalities or tracheostomy did not appear to be at greater risk of postoperative morbidity.^11^ Conversely, another report suggests that inadequate nutritional status increases the risk of urinary infections, prolonged patient intubation, as well as hospital stay.^29^ In the author's experience, poor preoperative respiratory function manifested with recurrent chest infections and inadequate secretion clearance can increase significantly the risk of postoperative complications, as well as the need for prolonged non-invasive ventilation. Poor nutritional state prior to surgery can affect wound healing and increase the risk of wound infection, which may in turn jeopardize the surgical outcome.

## LIFE EXPECTANCY FOLLOWING SPINAL SURGERY / QUALITY OF LIFE ASSESSMENT

Predicting life expectancy for children and adolescents with CP is difficult. Affected individuals were previously assumed to have lower survival rates compared to the general population.^60^ Significantly better survival rates even for the total-body involved patients have been recently documented and this reflects the improved medical support provided by the modern health systems.^61^,^64^

A relatively long mean predicted survivorship of 11.2 years has been reported in children and adolescents with severe spastic CP and neuromuscular scoliosis who underwent surgical correction.^31^ In the same study, the most accurate determinant for survival rates was the number of days the patient stayed postoperatively in ICU, which is primarily related to pulmonary complications and reflects the preoperative general medical condition of each patient.

Recent quality of life assessment studies in large populations of CP patients have demonstrated a very positive impact of spinal deformity correction with the vast majority of parents (95.8%) and caregivers (84.3%) who were interviewed considering that the benefits of spinal deformity surgery far outweigh any possible risks.^32^,^65^

## CONCLUSION

The development of spinal deformity is very common in patients with CP with prevalence proportionate to the degree of neurological involvement. The deformity can cause back and rib pain, as well as affect the patients' sitting balance and ability to function. Surgical correction and instrumented fusion is the only effective treatment and this has a positive impact on the quality of life and nursing care. Spinal surgery in patients with neurological disorders, multiple medical co-morbidities and extensive spinopelvic deformities is associated with a significant risk of severe and often life-threatening perioperative complications and should be performed by highly trained surgeons in specialized centers with adequate medical support. A prolonged postoperative recovery should be expected and it is important that the parents and caregivers fully understand and accept the risks involved with the surgery, as well as the anticipated benefits. With improved medical management and a multidisciplinary approach, life expectancy for this group of patients is much higher than previously expected and spinal fusion is the only surgical procedure that has such a high satisfaction rate among parents and caregivers, especially for quadriplegic patients.
